# Using the Electronic Medical Record to Examine Racial and Ethnic Differences in Depression Diagnosis and Treatment in a Primary Care Population

**DOI:** 10.4172/2167-1079.1000106

**Published:** 2012-01-04

**Authors:** Nhi-Ha T. Trinh, Rachel LaRocca, Susan Regan, Trina E. Chang, Stephen E. Gilman, Maurizio Fava, Albert Yeung

**Affiliations:** 1 Depression Clinical & Research Program, Massachusetts General Hospital, One Bowdoin Square, 6th floor, Boston, MA 02114, USA; 2 Division of Internal Medicine, Massachusetts General Hospital, 50 Staniford Street, 9th Floor, Boston, MA 02114, USA; 3 Department of Society, Human Development, and Health, Harvard School of Public Health, 677 Huntington Avenue Boston, MA 02115, USA; 4 Department of Epidemiology, Harvard School of Public Health, 677 Huntington Avenue Boston, MA 02115, USA; 5 Department of Psychiatry, Massachusetts General Hospital, 55 Fruit Street Boston MA 02114, USA; 6 South Cove Community Health Center, 885 Washington St Boston, MA 02111, USA

**Keywords:** Major Depressive Disorders, Minorities, Electronic Medical Records, Primary Care

## Abstract

**Objective:**

We assessed racial and ethnic differences in depression diagnosis and treatment in a primary care population.

**Methods:**

A sample of primary care outpatients in 2007 was generated using the electronic medical record (EMR). Patients were considered depressed if their providers billed for depression-related codes; they were considered prescribed antidepressants if any antidepressants were on their medication list. Rates of diagnosis and medication prescription were estimated using a generalized linear model with a Poisson distribution, adjusting for covariates.

**Results:**

In the resulting sample (n=85,790), all minority groups were less likely to be diagnosed with depression as compared to Whites (p<0.05); 11.36% of Whites had a depression diagnosis, as compared to 6.44% of Asian Americans, 7.55% of African Americans, and 10.18% of Latino Americans. Among those with a depression diagnosis (n=11,096), 54.07% of African Americans were prescribed antidepressant medications, as compared to 63.19% Whites (p<0.05); Asian Americans and Latino Americans showed a trend of being less likely to be prescribed antidepressant medications.

**Conclusions:**

Our study illustrates differences in diagnosis and treatment for minority primary care patients, and is innovative in using the EMR to probe these differences. Further research is needed to understand the underlying reasons for these observed differences.

## Introduction

Although depression can be reliably diagnosed and treated in primary care settings, according to the United States Surgeon General’s report on mental health in 1999, fewer than 25% of those affected are able to access clinically effective treatments, and the variability in accessibility is greater for ethnic and racial minorities [1]. Furthermore, depressive disorders are under-recognized and under-treated in these primary care minority populations [2-6].

Despite the similarities in prevalence of depression, minorities have been found to be less likely to be treated for depressive disorders in primary care settings. For example, Mexicans with major depressive disorder (MDD) have been found to be less likely than non-Hispanic Whites to receive antidepressant treatment [2], Mexicans and African Americans have been found to be significantly less likely than non-Hispanic Whites to use psychotherapy or pharmacotherapy to treat depressive symptoms [3], while non-Hispanic Whites have been found to use more antidepressants than any other race for any mood disorder [7,8]. In addition, African Americans and Caribbean Blacks who use antidepressants meet nationally-recognized treatment standards for major depression significantly less frequently than non-Hispanic Whites [3]. Taken together, these studies suggest a pattern of under-treatment of depression in minorities. However, these studies to date have relied on patient self-report of their depressive symptoms and treatment, rather than on clinician-based data, to estimate prevalence of diagnoses and treatments in these populations.

In this study, we used retrospective administrative and clinical electronic medical records (EMR) data from a primary care population of a large general hospital to explore and assess racial and ethnic differences in diagnosis and treatment for depressed patients. We hypothesized that we would find 1) lower percentages of reported clinical depression diagnoses in minority populations as compared to the White population. Furthermore, for those patients with a depression diagnosis, we hypothesized that we would find 2) lower percentages of reported psychopharmacological treatment in the minority populations as compared to the White population. This study is unique in that it examines a diverse, primary care population of a large general hospital, and uses EMR clinician-based data to probe discrepancies between diagnosis and treatment of depressive disorders.

## Methods

### Definition of race and ethnicity

In comparison to the White population, three broadly defined minority groupings were used to describe the study population by race and ethnicity: African American (Black), Asian/Pacific Islander, and Latino American (Hispanic). If subjects’ demographic information regarding race was missing, they were included in the “Other/Unknown” category. Native American/American Indian subjects were also included in the “Other/Unknown” category given their small numbers (n=65) relative to our overall sample. Data regarding race and ethnicity were gathered from patient self-report at hospital registration. Registration staff members at this hospital have been trained specifically to gather race and ethnicity data; data entry includes choices from a drop-down menu as well as free-text fields. Questions regarding race, ethnicity, and language are as follows: 1) regarding race: “Which of the following best describes your race/ethnicity? Please report all that apply”; 2) regarding ethnic origin: “Please tell us more about your ethnic origin. For example, are you (examples of ethnicity categories are given)? Please report all that apply”; and 3) regarding language, “What language do you prefer to speak? Please report only one.” All new patients are asked these questions during initial hospital registration, and existing patients are asked these questions once a year during registration verification; interpreters are used if needed.

### Study sample

We used the Research Patient Data Registry (RPDR), the hospital research data warehouse synthesizing EMR data from a variety of administrative and clinical databases; through the RPDR, investigators can generate a patient database of interest using specific criteria [9,10]. In June 2008, we requested data from all primary care outpatients age 18 or older seen in January-December 2007 at this hospital. Patients were included in the study sample if they: 1) were outpatients seen in the primary care practices at our hospital, 2) lived in Massachusetts and the states of Connecticut, New York, Rhode Island, New Hampshire, Vermont, or Maine (based on zip code), and 3) had least one outpatient encounter with their primary care physician at this hospital in 2007, using an algorithm described previously [11]. Subjects were excluded from the data sample if: 1) their billing encounters were missing a physician or principal diagnosis and thus could not be categorized, or 2) if they died during 2007. Data from encounters coded as labs or from pharmacy were also excluded.

### Definition of depression diagnosis and antidepressant use

Using a previously validated algorithm, billing diagnoses from the EMR were used to categorize patients as having clinical depression [12]. Patients were considered depressed if any of their providers billed for any of a variety of depression-related codes in the year 2007. This data source has been shown in our sample to reflect primary care physician diagnostic impression of clinical depression with 77% sensitivity, 76% specificity, and area under receiver operating characteristic curve of 0.77 [12]. Although the depression-related codes included are quite broad, using this “billing diagnosis” data source was found to be the most sensitive as compared to other fields in the EMR. Regarding medication use, patients with a billing diagnosis of depression were considered prescribed antidepressants for depression if any antidepressants were included in their medication list.

### Definition of socioeconomic status

Subject socioeconomic status was estimated using median income from 2000 U.S. Census data aggregated at the census tract level [13]. Using census tract level data, which breaks down geographical areas defined by zip code into smaller areas called tracts to estimate median income, has been shown to be more sensitive to socioeconomic gradients in health than using median income estimated by zip code alone [14]. Mapmarker software, which matches identified street addresses to corresponding census tracts, was used to identify census tracts for patients in the sample [15]; the census tract data generated was then used to generate median income from US census data. Successful tract-level identifications were made for 91% of patients.

Town-, zip code-, or county-level data were used for the remaining 9%. Forty-five patients were excluded from the analysis because no valid address was available. Income was then divided into quintiles for use in the statistical model below.

### Statistical analysis

We estimated rates of diagnosis and prescription of medication by race using a generalized linear model with a Poisson distribution and log link, adjusting for patient gender, age, marital status, use of English as a primary language, estimated outcome, number of outpatient encounters (divided in quartiles), and having a hospitalization at this hospital during 2007; confidence intervals were estimated using robust standard errors [16]. Analyses were performed using Stata statistical software [17]. All study procedures were approved by the hospital Institutional Review Board.

## Results

Using the criteria above, 86,859 patients had at least one outpatient encounter with their primary care provider in 2007. Of these patients, 1000 did not live in the six states listed above, 45 patients were excluded because no valid address was available, 24 patients were excluded because their encounters were all inpatient, pharmacy or lab encounters, and a total of 65 Native American/American Indian subjects were included in the race “Other/Unknown” group. The resulting sample of 85,790 patients consisted of 80% Whites, 5% Asian Americans, 5% African Americans, 8% Latino Americans, and 3% listed as Other/Unknown; the demographics of the study sample, including age, gender, marital status, primary language, and median income are described in Table 1. The numbers and percentages of patients with a depression diagnosis and patients prescribed antidepressant medications are also described in Table 1. The differences between the excluded group (n=1069) and the rest of the sample (n=85,790) were not examined given that the excluded group made up only 1% of the original sample.

A multivariate model was constructed with the following covariates: gender, age, marital status, use of English as a primary language, estimated income, number of outpatient encounters in 2007, and having been hospitalized at this hospital during 2007. In this model, the following covariates were statistically significant: gender, age, marital status, estimated income, number of outpatient encounters, and having been hospitalized at this hospital in 2007 (Table 2). Using this multivariate model, all minority groups (Asian American, African American, Latino American) were less likely to be diagnosed with depression as compared to the White group (p<0.05) (Table 2). In our sample, adjusting for the above covariates, 11.36% of Whites had a depression diagnosis, as compared to 6.44% of Asian Americans, 7.55% of African Americans, and 10.18% of Latino Americans. Interestingly, among those individuals with a depression diagnosis (n=11,096), 54.07% of African Americans were prescribed antidepressant medications, as compared to 63.19% Whites, a result that was statistically significant (p<0.05). For Asian Americans and Latino Americans with a depression diagnosis, these groups showed a trend of being less likely to be prescribed antidepressant medications (57.38% and 61.68% respectively) as compared to Whites, a result that was not statistically significant.

## Discussion

Our results indicate that minority primary care populations were less likely to receive depression diagnoses than the White primary care population, in multivariate analyses controlling for gender, age, marital status, use of English as a primary language, estimated income, number of outpatient encounters, and having been hospitalized at this hospital in 2007. Furthermore, we found that among those patients with a depression diagnosis, African American patients were significantly less likely to be prescribed antidepressant medications as compared to Whites; a similar trend was found for Asian Americans and Latino Americans.

Minority primary care populations in this sample were less likely to receive a depression diagnosis than the White primary care population, which may reflect under-diagnosis of depression in minorities as compared to Whites. However, that this finding may also reflect differences in true disorder rates between groups cannot be definitively excluded. Precise estimates of the 12-month prevalence of depressive diagnoses in minority populations for population-based epidemiological studies have been difficult to achieve, given the diversity of ethnic backgrounds and languages of target populations. Recent analyses using the Collaborative Psychiatric Epidemiology Survey (CPES) have used pooled data from three population-based epidemiology studies of depressive disorders to estimate 12-month prevalence of dysthymia and MDD as 11.2 % for Whites, as compared to 10.8% for Latino Americans, 8.0% for African Americans and 5.4% for Asian Americans, and a more recent analysis of CPES data focusing on 12-month prevalence for MDD alone showed a similar pattern of lower prevalence of MDD in minorities as compared to Whites [18,19]; these 12-month prevalence rates are comparable to the prevalence rates found in our study. Future studies will need to explore whether minority primary care populations are less likely to receive a depression diagnosis than the white primary care population due to clinical under-diagnosis versus underlying differences in true disorder rates between groups.

In our study, among those with a depression diagnosis, African American primary care outpatients were significantly less likely to be prescribed antidepressant medications as compared to Whites; a similar trend was found for Asian Americans and Latino Americans. These findings are important as they replicate earlier research findings demonstrating differences in treatment for minorities [2,4]. An analysis of the CPES found African Americans to be significantly less likely to use pharmacological treatments for depression than Whites [3]. Asian Americans have been shown to have a pattern of underutilization of mental health services compared to other ethnic groups, characterized by delayed onset of treatment and higher attrition rates [22]. Latino Americans may have had fewer helpful mental health treatment experiences than non-Latino Whites, which may make them less willing to seek treatment in the future [23]. Finally, an analysis of the NCS-R found Whites were significantly more likely to use antidepressants than any other racial or ethnic group [8].

Our use of the EMR to investigate differences in depression diagnosis and treatment patterns is innovative. Instead of using patient self-report, our study focused on EMR clinician-based data on diagnosis and treatment. Data from self-report surveys may be subject to incomplete information, particularly if patients were unaware of their depression diagnosis or if they were being treated for depression. Many from ethnic and racial minority populations are less likely to discuss their treatment with their primary care providers and may be unaware that they are being treated for depression [24]. To our knowledge, this is the first study to employ clinician-based data from the EMR to systematically quantify rates of depression diagnosis and treatment. Our method of using the EMR to estimate rates of diagnosis in specific patient populations can serve as a model for other primary care settings.

A variety of provider-based, patient-based, or systems-based factors can contribute to the under-diagnosis and treatment of depression in minority primary care populations. Primary care providers evaluating patients with a different cultural background or speaking a different language may have difficulty assessing depressive symptoms in minority populations, who may present with somatic or atypical complaints [25-27]. In addition, patient factors including the perceived availability of services, mistrust of mental health professionals, and stigma towards mental illness can prevent individuals from reporting psychiatric symptoms or pursuing mental health treatment [28-30]. Even if mental health care were universally available, there may still remain differences in treatment preferences, often due to cultural beliefs and values. For example, some African Americans are more likely to access mental health care in primary care settings than traditional mental health settings [31-33]; some consider prescription medications less acceptable and spiritual counseling more socially acceptable compared to Whites [29], all of which may decrease the likelihood of either being diagnosed with depression or prescribed antidepressants. Thus a variety of factors may contribute to the differences in diagnosis and treatment observed in this study.

Certain limitations to the study are worth noting. We used billing codes from the EMR as a proxy for a clinical diagnosis of depression. Although we validated this method as having moderate accuracy in this hospital population, using EMR billing codes still remains an estimate for the gold standard of a detailed face-to-face clinician interview and assessment [12]. However, employing this method with a very large sample size of outpatients provided us a snapshot of the general trends and differences in this primary care setting. For this analysis, we did not explore the role of medical co-morbidity in relationship to the diagnosis and treatment of depression. We did, however, account for some degree of significant co-morbidity by controlling for the number of inpatient hospitalizations the study sample had during this time period. Finally, a potential limitation of this database was that it was not able to capture referrals for or receipt of psychotherapy, thus full patterns of treatment differences among racial and ethnic populations were not defined.

Exploring what differences exist for minority primary care populations in depression diagnosis and care is only the first step; we must also understand the underlying factors behind these differences. Future studies in this area should focus on understanding reasons for observed differences in prevalence rates of depressive disorders, exploring missed opportunities in the clinical encounter, identifying the personal and cultural barriers to care in minority groups, and examining and combating the systemic sources of under-treatment in these groups. Identifying which populations are affected is the first step to the ultimate goal: creating innovative and effective interventions to increase access to care in minority primary care populations.

## Conclusion

A number of studies to date have explored differences in mental health treatment in minority populations. Our analysis sought to examine differences in depression diagnosis and treatment in a primary care setting of a large general hospital. Our results indicated that minority primary care populations had lower rates of depression diagnoses than the White primary care population; in addition, for those diagnosed with depression, African Americans in particular were less likely to receive antidepressant medications as compared to the White primary care population. Our analysis is innovative in using the EMR to probe differences in rates of diagnosis and treatment in minority primary care populations and serves as a first step in understanding and addressing these differences.

## Figures and Tables

**Table 1:** Description of Patient Sample.

	White	Asian American	African American	Latino American	Other/Unknown	TOTAL
Patients, n (% total)	68,401 (80)	4,010 (5)	3,950 (5)	7,144 (8)	2,285 (3)	85,790 (100)
Outpatient Encounters total (median)	722,813 (7)	32,650 (6)	42,990 (7)	80,775 (8)	18,958 (5)	898,186 (7)
Age, mean (SD)	53 (17.1)	46 (16.1)	48 (15.8)	43 (14.4)	48 (16.4)	52 (17.1)
Women, n (%)	39,941 (58)	2,658 (66)	2,496 (63)	4,682 (66)	1,275 (56)	51,052 (60)
Married or Living with Partner, n (%)	40,021 (59)	2,568 (64)	1,780 (45)	3,036 (43)	1,301 (60)	48,706 (57)
English-speaking, n (%)	67,413 (99)	2,974 (74)	3,632 (92)	2,516 (35)	1,965 (86)	78,500 (92)
Hospitalized, n (%)	4,909 (7)	206 (5)	322 (8)	547 (8)	130 (6)	6,114 (7)
Median income (US dollars)	58,523	48,991	41,771	32,134	51,352	54,125
Patients with depression diagnosis, n (%)*	9038 (13)	283 (7)	421 (11)	1119 (16)	235 (10)	11,096 (13)
Of patients with depression diagnosis, patients prescribed medication, n (%)*	6210 (69)	176 (62)	265 (63)	824 (74)	150 (64)	7,625 (69)

* Percentages here are unadjusted. Percentages for each minority population category compared to the White population (adjusted for covariates) are described in the text.

**Table 2:** Multivariate Models Predicting Rate of Depression Diagnosis.

		**Model predicting Depression Diagnosis** n=85,790 df= 85,778			**Model predicting Medication Prescription** *(for patients with a depression diagnosis)*n=11,096 df=11,084		
		Adjusted Rate Ratio^1^	P	95% CI	Adjusted Rate Ratio^1^	P	95% CI
Race	White	1.00			1.00		
	Asian American	0.57*	<0.001	0.51-0.64	0.91	0.044	0.83-1.00
	African American	0.66*	<0.001	0.61-0.73	0.86*	<0.001	0.80-0.92
	Latino American	0.90*	<0.001	0.83-0.97	0.98	0.361	0.93-1.03
	Unknown/Other	0.84*	<0.001	0.75-0.95	0.91	0.064	0.83-1.01
Female gender		1.38*	<0.001	1.33-1.44	1.10*	<0.001	1.07-1.13
Age (in decades)		0.95*	<0.001	0.94-0.96	0.97*	<0.001	0.97-0.98
Married		0.70*	<0.001	0.68-0.73	1.02	0.191	0.99-1.04
Primary language English		1.03	0.716	0.95-1.12	1.02	0.545	0.96-1.07
Income (quintiles)		0.94*	<0.001	0.92-0.95	0.97*	<0.001	0.96-0.98
Number of encounters (quartiles)		1.54*	<0.001	1.51-1.57	1.13*	<0.001	1.11-1.15
Hospitalized as inpatient in 2007		1.39*	<0.001	1.33-1.46	1.09	<0.001	1.06-1.12

1 Adjusted for other factors in the model: gender, age, marital status, use of English as a primary language, estimated income, number of outpatient encounters in 2007, and having been hospitalized at this hospital during 2007.

* p<0.05
